# Post-fire insect fauna explored by crown fermental traps in forests of the European Russia

**DOI:** 10.1038/s41598-021-00816-3

**Published:** 2021-10-29

**Authors:** A. B. Ruchin, L. V. Egorov, I. MacGowan, V. N. Makarkin, A. V. Antropov, N. G. Gornostaev, A. A. Khapugin, L. Dvořák, M. N. Esin

**Affiliations:** 1 Mordovia State Nature Reserve and National Park “Smolny”, Saransk, Russia; 2 Prisursky State Nature Reserve, Cheboksary, Russia; 3 National Museums of Scotland, Collection Centre, Edinburgh, Scotland, UK; 4 Federal Scientific Center of the East Asia Terrestrial Biodiversity, Far Eastern Branch of the Russian Academy of Sciences, Vladivostok, Russia; 5 Zoological Museum, Moscow State University, Moscow, Russia 125009; 6 N.K. Koltsov Institute of Developmental Biology RAS, Moscow, Russia; 7 Tyumen State University, Tyumen, Russia; 8 Tři Sekery, Mariánské Lázně, Czech Republic

**Keywords:** Biodiversity, Fire ecology

## Abstract

Wildfires considerably affect forest ecosystems. However, there is a lack of data on the post-fire status of insect communities in these ecosystems. This paper presents results of a study conducted in 2019 which considered the post-fire status of the insect fauna in a Protected Area, Mordovia State Nature Reserve (Republic of Mordovia, centre of European Russia), considered as regional hotspot of insect diversity in Mordovia. We sampled insects on intact (unburned, control) and fire-damaged (burnt in 2010) sites and compared the alpha-diversity between sites. In total, we sampled and analysed 16,861 specimens belonging to 11 insect orders, 51 families and 190 species. The largest orders represented in the samples were Coleoptera (95 species), Diptera (54 species), Hymenoptera (21 species), and Neuroptera (11 species). Other insect orders were represented by between one and four species. The largest four orders (Coleoptera, Lepidoptera, Diptera and Hymenoptera) represented 96.7% of all studied specimens. We found that in the ninth year after low intensity surface fire damage, the insect diversity had returned to a similar level to that of the control (unburned) sites. Sites damaged by crown wildfire differed considerably from other sites in terms of a negative impact on both species diversity and the number of specimens. This indicates the serious effect of the crown fires on the biodiversity and consequent long-term recovery of the damaged ecosystem.

## Introduction

Wildfires are one of the main factors influencing natural ecosystems around the world. They have a variety of effects on the environment and the Earth’s climate system^1–3^. Wildfires are a part of the forest ecosystem evolution and the status of forest vegetation. However, it is unclear how frequent and severe wildfires affect the interaction of species and ecological and evolutionary processes and there is an obvious need for experimental studies^4^.

In Russia, the role of wildfires in natural ecosystems is twofold. Firstly, in unmanaged forests at high latitudes, characterised by a small human population density, ground wildfires are a part of the natural forest cycle Such wildfires prevent a decrease in forest productivity, the waterlogging of soils, and the spread of “green desertification”^5–9^. Secondly, in less boreal and more densely populated areas, wildfires are a very harmful natural disturbance event. Such fires determine forest succession, the mosaicity and structure of the forest cover and the quantitative and qualitative characteristics of forest stands and lead to significant ecological, economic and social losses. Wildfires were especially serious in European Russia during 2010 where they had a considerable impact on many forest ecosystems^10–14^.

The consequences of a wildfire for animals can be direct and immediate, although some of the consequences (e.g. shortening of life expectancy or deterioration in physiological state) may take years or decades to become evident. The response of invertebrates to wildfires is the result of both direct, immediate impact of fire and longer-term post-fire changes to the ecosystem. The direct impact of a wildfire depends on the fire type, as well as on the biotic conditions prevailing within the forest. For insects important factors are the stage of development of the species and its mobility during the wildfire event^15,16^. The impact of wildfire on forest ecosystems largely depends on the area burnt, fire type (e.g. crown fire vs. surface fire) and intensity of the fire. The most severe fires, usually crown fires, change the composition of the forest stand and affect all components of the forest ecosystem, including the nutrient cycle^17–19^.

In terms of wildfire impacts on the insect fauna most of the literature considers mainly the impact on soil-dwelling and terrestrial arthropods during a 10–15-year period after the wildfire^4,20–24^. Post-fire changes in both litter and soil depend considerably on the intensity and duration of the wildfire, as well as on other factors^25–27^. The loss of the litter and upper organic soil layer is an important negative factor for soil-dwelling animals^28^. In contrast, other literature sources demonstrate the positive response of saproxylic insects after a wildfire event^29–33^. Only a few publications have studied the post-fire changes in the arthropod fauna including actively mobile insects such as Diptera and Hymenoptera^34–41^. The present paper seeks to identify the impact of a 2010 wildfire on the insect fauna in forests of the Mordovia State Nature Reserve.

## Materials and methods

### Study area

**Figure 1:**
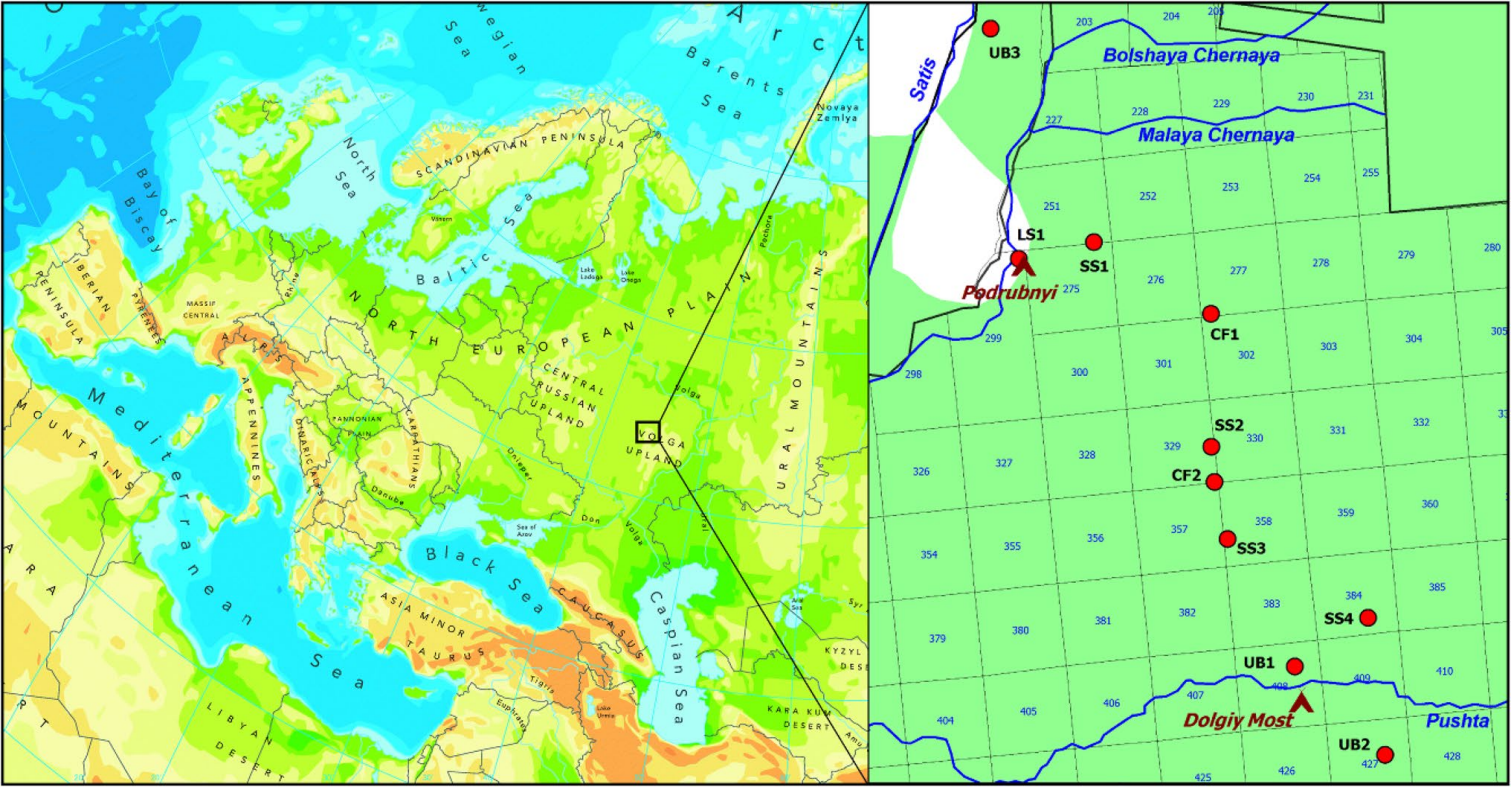
Geographical position of the Mordovia State Nature Reserve in Europe. Study plots are named according to designations in Table 1. Mordovia State Nature Reserve’s forest compartments are numbered. The map has been created using the MapInfo 11.5 software. Map with modifications from https://www.eea.europa.eu/data-and-maps/figures/physical-map-of-eurasia.

The study was carried out in the Mordovia State Nature Reserve (European Russia), located in the southern boundary of the taiga zone (54° 42′–54° 56′ N 43° 04′–43° 36′ E; up to 190 m a.s.l., Fig. 1). The Mordovia State Nature Reserve contains natural ecosystems in centre of European Russia acknowledged as a hotspot for biodiversity^42,43^. The total area of the Protected Area is 321.62 km^2^ with forest communities covering 89.3% of this area. Pine (*Pinus sylvestris* L.) is the main forest tree species where it forms pure or mixed forest communities. Birch (*Betula pendula* Roth) is the second commonest tree species and forms predominantly secondary forest communities on old logging or burnt areas. Small-leaved linden (*Tilia cordata* Mill.) forms pure stands in the northern part of the Mordovia State Nature Reserve, as well as being important in the development of an undergrowth layer in pine stands and mixed forests. Oak (*Quercus robur* L.) forests occupy relatively small areas mainly on the floodplain of the Moksha River in the western part of the Mordovia State Nature Reserve. Spruce (*Picea abies* L.) forests are also located predominantly on river floodplains (Pushta, Vyaz-Pushta, Vorsklyay, Arga, etc.) and cover small areas. There are numerous oligotrophic mires dominated by *Sphagnum* or *Sphagnum—Carex* communities. Floodplain meadows are situated mainly in floodplains of Satis and Moksha Rivers in western and northwestern sites of the Protected Area^44^. Soils are classified as predominantly sand in varying degree of podzolisation. These lie on the ancient alluvial sands. Sandy peaty podzolic soils are also widely spread on sands with a fairly high level of ground water. Sandy podzolised soils are located under deciduous forests. Easily loamy soils are distributed in same conditions but much less frequently. The mean annual precipitation is 406.6–681.3 mm. The mean annual air temperature is 4.7 °C. Maximal values are registered in July, and minimal values in February^45^. In the Mordovia State Nature Reserve, serious wildfires observed in 1842, 1899, 1932, 1972, 2010, and 2019^24,46–48^. In 2010, the wildfires were especially serious with approximately 38% of the total area of the Mordovia State Nature Reserve being affected. At the same time, the degree of intensity and severity of the wildfire varied at various sites of the Protected Area^49^.

### Sampling and identification

Most of the collected insects were identified by the authors to species level. Additionally, Diptera and other specialists were involved in the identification process. As a result of processing the material, 16,861 specimens were examined. Insect samples are classified according to Fauna Europaea (https://fauna-eu.org). We also consulted the latest lists of individual groups of insects^50^. The order of taxa names within families is alphabetical. Years of description of some species are specified by^51^).

Field samples were collected using crown traps located at a height of 6–7 m above the ground. Detailed description of a crown trap design follows^52^. We established one crown trap per habitat. All measurements were replicated seven times from 02 June to 15 August 2019.

The study sites were characterised in terms of the habitat illumination, distance from the edge of burned area and wildfire severity and intensity (Table 1). Habitat illumination was assessed visually. Fire severity was estimated according to Ryan^53^ and Turner et al.^54^ with modifications. Evaluation of the fire intensity was carried out according to the Fire Intensity Risk System^55^. In addition, we estimated its strength using 100-score scale.

**Table 1:** Characteristics of the 2010 wildfire for each study plot.

Plots	Habitat illumination	Distance from the edge of burned area, km*	Severity	Intensity
SS1	60	− 1.8	Severe surface fire	Moderately vigorous surface fire (40)
LS1	50	− 0.3	Light surface fire	Low vigorous surface fire (10)
CF1	100	− 4.0	Crown fire	Extremely vigorous surface fire or active crown fire (100)
CF2	100	− 4.6	Crown fire	Extremely vigorous surface fire or active crown fire (100)
SS2	50	− 5.0	Severe surface fire	Moderately vigorous surface fire (40)
SS3	75	0	Severe surface fire	Moderately vigorous surface fire (25)
SS4	75	0	Severe surface fire	Moderately vigorous surface fire (35)
UB1	50	+ 1.0	Unburned	Unburned (0)
UB2	50	+ 2.3	Unburned	Unburned (0)
UB3	30	+ 1.5	Unburned	Unburned (0)

*Minus sign indicates that a site is within the burned area; plus sign indicates that a site is outside of the burned area.

In total, we used ten study plots (100 m × 100 m). Three were control, unburned sites square 408 (UB1), square 427 (UB2), and 0.8 of the Protected Area border (UB3)) two were damaged by an extremely vigorous crown fire (CF1, CF2), one burned by a light surface fire (LS1), and four damaged by severe surface fire (SS1, SS2, SS3, SS4) (Appendix A). Species composition and structure of post-fire communities were similar to vegetation samples studied by Khapugin et al.^49^ in similar conditions.

### Statistical analysis

We calculated three widely-used biodiversity indices, namely the Margalef index^56^, Shannon index^57^, and the Simpson species evenness index^58^, for each plot studied. To compare species composition between studied plots we used the Euclidean distance. We did not consider insects which were not identified to species level.

$$H = - \sum\limits_{i = 1}^{S} {\frac{{n_{i} }}{N}\ln \frac{{n_{i} }}{N}}$$

where *H* is the diversity in a study plot of *S* species, *n*_*i*_ is the number of individuals of the *i*th species, *N* is the total number of individuals of all the species and ln is the natural logarithm. The higher value of *H* means higher species richness and also signifying that different species in the quadrat or a community are nearly equally abundant.

The Simpson species evenness index (D) used in this study is given by the following formula:

$$D = 1 - \sum\limits_{i = 1}^{S} {\left( {\frac{{n_{i} }}{N}} \right)^{2} }$$

*D* profits into justification both the number of species and the equilibrium among them. The value of *D* falls within the interval [0…1] if there is only one species, *D* is zero. As the number of species increases (and their contribution to overall abundance is equalised) *D* approaches 1.

To analyse and visualise the relationships between used characteristics of habitats (habitat illumination; distance from the edge of burned area; wildfire intensity) and caught index, we used Canonical correspondence analysis (CCA). Statistical analyses were carried out using PAST^59^ and Microsoft Excel.

## Ethics approval and consent to participate

Our study was conducted in compliance with the ethical standards of humane treatment of animals in accordance with the recommended standards described by the Directive of the European Parliament and of the Council of the European Union of 22 September 2010 "On the protection of animals used for scientific purposes" (EU Directive 2010/63/EU).

## Results

### Insect fauna

**Figure 2:**
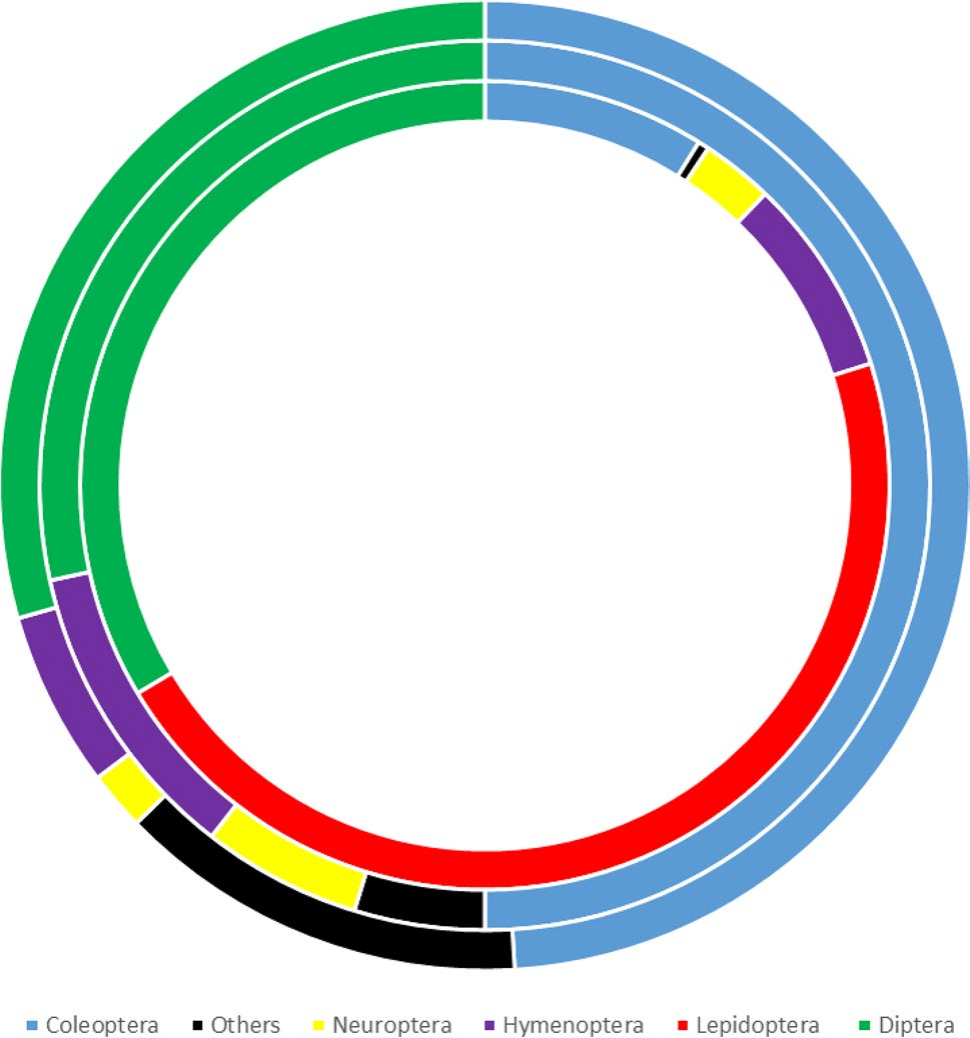
Taxonomic composition and ratio of the insect orders caught using crown traps. *Note*: Category «Others» includes the following orders: Dictyoptera, Dermaptera, Heteroptera, Raphidioptera, Mecoptera, Trichoptera. The inner ring is the number of specimens. The middle ring is the number of species. The outer ring is the number of families.

We identified 190 species from 51 families (Appendix B) and 11 orders (Fig. 2). Due to the poor quality of some of the collected material some Lepidoptera, Trichoptera, and particularly Coleoptera and Diptera could not be identified to either species or genus level. Four orders, Coleoptera, Lepidoptera, Diptera and Hymenoptera, represented 96.7% of the sampled specimens.

The order Coleoptera was represented by 95 species from 25 families. The highest taxonomic diversity was found for Cerambycidae (22 species), Elateridae (11 species), Nitidulidae (8 species), Curculionidae (8 species), and Scarabaeidae (7 species). Among Coleoptera, the highest number of specimens caught by the crown traps was found for Cerambycidae, Nitidulidae and Scarabaeidae which combined represented 81.2% of the entire number of insect specimens.

Lepidoptera—unfortunately, the quality of specimens caught in crown traps was poor and as a result we were able only to estimate the number of specimens collected in various study plots. We found this number to be highly variable depending on the study plot. In plots CF1 (361 specimens) and CF2 (182 specimens) the number of specimens was lowest whilst the greatest number of specimens was found in plots UB3 (1762 specimens) and UB1 (1245 specimens). The intermediate values were revealed in the study plots located at the edge of the burned area.

The order Hymenoptera was represented by 21 species belonging to three families. The order Diptera captures included 54 species from 15 families. The highest taxonomic diversity was found for the families Drosophilidae (15 species), Muscidae (10 species), and Lonchaeidae (8 species). The same families also dominated in terms of the number of specimens. Eleven species of Neuroptera were identified during the study period. Other insect orders were only represented by a few numbers of species with small number of specimens.

### Analysis of insect distribution in the study area

**Figure 3:**
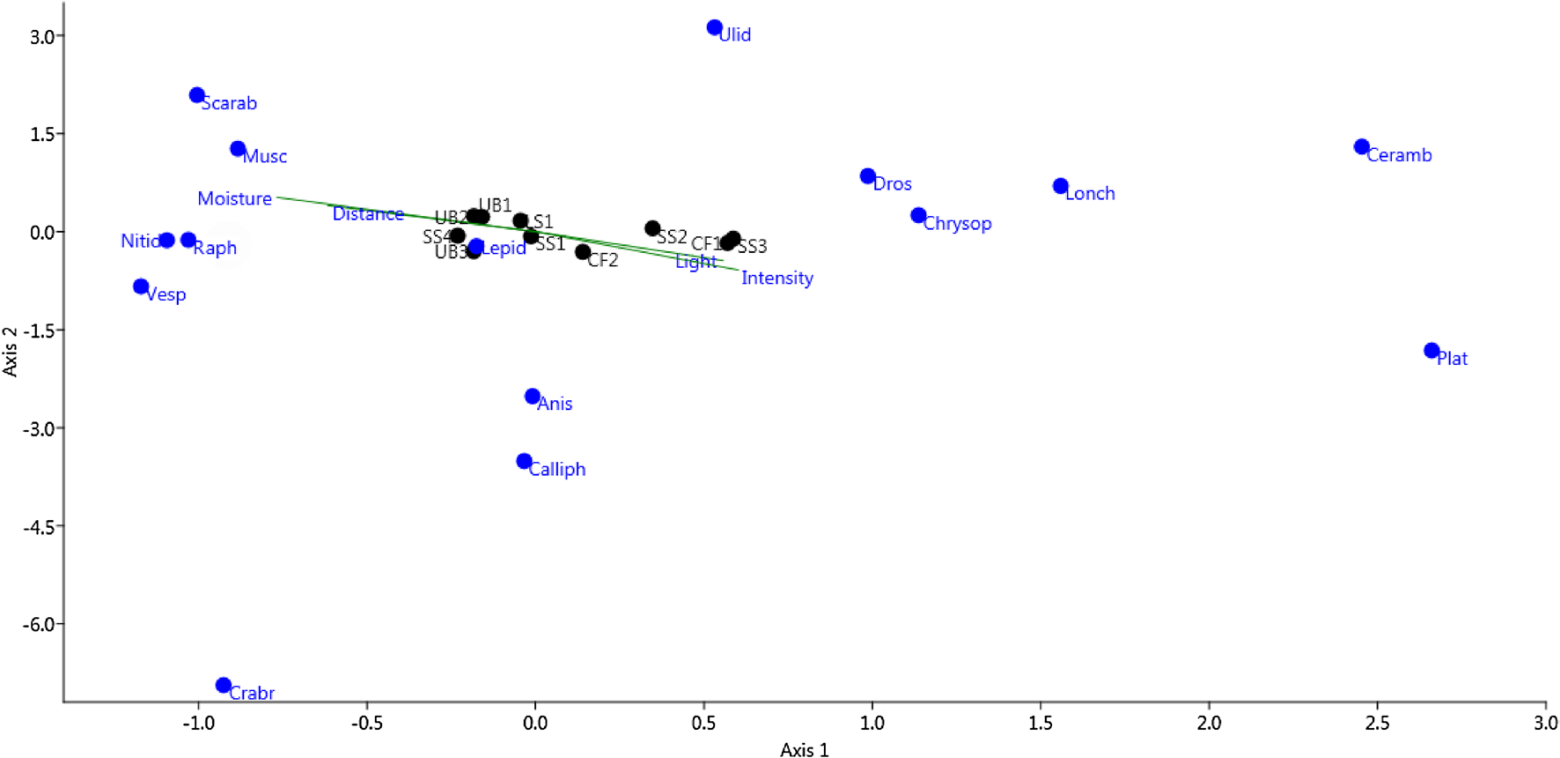
The canonical correlation analysis of the number of the caught insect specimens depending on the habitat illumination (Light), distance from the edge of the burned area (Distance), fire intensity (Intensity), soil moisture (Moisture). Designations: order Coleoptera: Scarab—Scarabaeidae, Nitid—Nitidulidae, Ceramb—Cerambycidae; order Neuroptera: Chrysop—Chrysopidae; order Raphidioptera: Raph—Raphidiidae; Lepid—Lepidoptera; order Diptera: Musc—Muscidae, Poll—Pollenidae, Plat—Platystomatidae, Ulid—Ulidiidae, Lonch—Lonchaeidae, Anis—Anisopodidae, Dros—Drosophilidae; order Hymenoptera: Vesp—Vespidae, Crabr—Crabronidae.

The canonical correlation analysis (CCA) demonstrated that the habitat illumination and the fire intensity influenced at the same direction as it is shown from the study plot arrangement on the CCA plot (Fig. 3). So, the influences of these environment factors are correlated.

The increase in both habitat illumination and fire intensity correlates positively with the number of the caught Platystomatidae (Diptera) specimens. This is less pronounced for the following families: Cerambycidae (Coleoptera), Lonchaeidae, Pollenidae, Anisopodidae, Drosophilidae (Diptera), Chrysopidae (Neuroptera). However, the number of the caught Scarabaeidae (Coleoptera) decreases under these conditions.

The number of Vespidae (Hymenoptera), Nitidulidae and Scarabaeidae (Coleoptera), Muscidae (Diptera), and Raphidiidae (Raphidioptera) species correlates positively with soil moisture in the study sites. Increasing study plot distance from the burned area edge leads most significantly to an increase in the number of the Scarabaeidae specimens, this trend is present but less pronounced for Nitidulidae, Muscidae, Ulidiidae (Diptera) and Vespidae (Hymenoptera). Conversely, distance from the burn edge caused a decrease in the number of specimens of Platystomatidae and Cerambycidae captured. Distance from the burn edge had no apparent affect on the number of Crabronidae (Hymenoptera) specimens caught which perhaps indicates the lack of the environment factors’ influencing this insect group.

There were differences between study plots both in terms of the number of specimens and species caught. Traps in plot UB3 produced the greatest number of both specimens and species caught (Margalef index = 11.16). The lowest numbers were recorded in plots CF1 and CF2 (Margalef index = 7.21 and 8.13 respectively) damaged by vigorous crown fires (Table 2). This was confirmed by the Margalef index calculations.

**Table 2:** Biological diversity indexes calculated for the ten studied habitats.

	Plot SS1	Plot LS1	Plot CF1	Plot CF2	Plot SS2	Plot SS3	Plot SS4	Plot UB1	Plot UB2	Plot UB3
Margalef index	8.37	9.51	7.21	8.13	9.50	10.20	9.23	8.48	9.08	11.16
Shannon index	2.66	2.72	2.87	3.19	2.77	2.92	2.99	2.31	2.68	2.80
Simpson index	0.15	0.14	0.09	0.06	0.12	0.09	0.09	0.24	0.17	0.12
Number of species	55	71	43	41	64	66	57	59	61	81
Total number of specimens	1249	2835	736	335	1622	1096	1141	2380	1782	3685

The calculated Shannon index and Simpson index demonstrated the following results. In plot CF2, we found the maximal Shannon index values and minimal Simpson index values. The similar results were obtained in plot CF1. In both cases, this is caused by the evenly distribution of the insect species in terms of their abundance in catches. Therefore, despite the minimal number of the species caught in the plots damaged by the crown fires, their species diversity was characterised by the maximal evenness and minimal dominance of certain species. In the control (unburned) plot UB1, we found an inverse relationship, i.e. the lowest Shannon index and the highest Simpson index.

**Figure 4:**
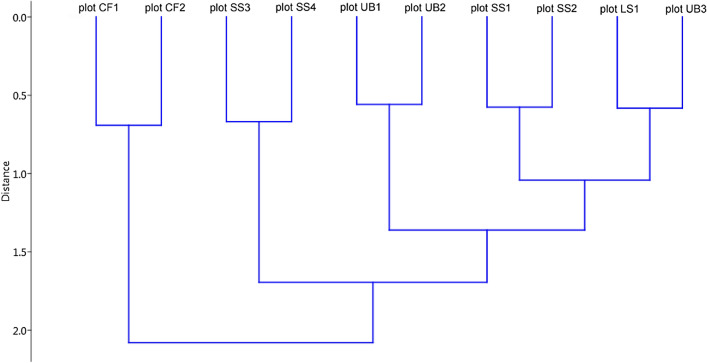
The Euclidean distances between the insect species composition for the ten studied habitats (Ward's hierarchical clustering; cophenetic correlation coefficient = 0.788).

The dendrogram based on Euclidean distances demonstrated the considerable differences between plots crown fire-damaged plots (CF1 and CF2) and other studied sites (Fig. 4). At the same time, the differences between plots CF1 and CF2 were minimal. The burned area edges (plot SS3 and plot SS4) were placed at the same cluster, with minimal differences in the species compositions.

There were small differences in the species diversity between plot SS1 and plot SS2. Both these sites are within 3 km each of other and the structures of their canopy and herb layer are highly similar. These two study plots are also similar in terms of the severity and intensity of the fire, which damaged them in 2010. Therefore, the insect faunas in these sites are quite similar. The comparison of both control (unburned) plots (UB1 and UB2) demonstrated the analogous results. These study plots are located into the natural intact ecosystems of the mixed forests in the Mordovia State Nature Reserve. In these study plots, the insect fauna is typical for the forest ecosystems in central European Russia. The similar species composition was registered in plot LS1 (damaged by the light surface fire) and in control (unburned) plot UB3. Like the control plot UB3, plot LS1 is located nearby of the small river. We conclude that in plot LS1 full post-fire restoration has occurred. Due to this, the restoration of insect species diversity in plot LS1 has returned to the level observed in the control plot UB3.

## Discussion

Makarkin and Ruchin^60^ noted that crown traps baited with beer attract only green lacewings (Chrysopidae), but not predatory species, and only those whose adults feed mainly on pollen and honeydew (i.e., phytophagous and glycophagous). These are species of the genera *Nothochrysa*, *Nineta*, *Apertochrysa*, *Chrysoptropia*, and *Chrysoperla*. Green lacewings of these genera are mainly dendrobionts living on various deciduous trees and shrubs (less often on pines), without any preference for tree genus or species. The only exception is *Nothochrysa fulviceps*, which prefers oak (*Quercus robur* in this region).

This knowledge makes the succession of the post-fire neuropteran assemblages more understandable. In general, the number of individuals and species is greatest where there are more deciduous trees (plot CF2, comparable to plot UB3), and least where there are few deciduous trees, or the shrub undergrowth is poorly developed (plot SS1, plot CF1, comparable to plot UB1). The high abundance of Chrysopidae in plot SS2 may be explained by the presence of dense shrub undergrowth.

The number of individuals of *Apertochrysa prasina*, *Chrysotropia ciliata*, and *Nineta alpicola* constitutes 82.7% of all captured Neuroptera. *A. prasina* prefers drier biotopes, and may be found both on shrubs and in tree canopies. This may explain the presence of this species at all sites. *Chrysotropia ciliata* is a hygrophilous species that prefers colder, shady and humid microclimatic conditions. It may be found in deciduous and mixed forests, those along streams, and in forest openings with an abundant herb layer^61–64^. This probably explains the largest number of captured specimens of this species in plots UB2 and UB3. In these plots, higher soil moisture was noted compared to other areas. *Nineta alpicola* inhabits similar, but not so humid, forests.

Forest edges are usually the hotspots for Chrysopidae species diversity^63^. It could be predicted that a higher species diversity of this family would be observed at the edge of the burned areas (plot SS3 and plot SS4) but our results do not confirm this. The largest number of Chrysopidae species was recorded in plot LS1 (8 species) and plot UB3 (7 species), where humid and dry habitats combined with a well-developed shrub layer. This is evidenced by the similarity of the species composition in plot LS1 and plot UB3.

The relatively high number of *Dichrostigma flavipes* (Raphidiidae) captured by crown traps is difficult to explain. All species of Raphidiidae are assumed to be predators, whereas adults of at least some Inocelliidae are assumed to feed on flower pollen and nectar^65^. It is possible that adults of this species of Raphidiidae also visit flowers for food. The larvae of *D. flavipes* are obligately terricolous. They live in the soil of both coniferous and deciduous forests, in contrast to all other our species, whose larvae live on trees (mainly under the bark)^66,67^.

The highest abundance of Nitidulidae specimens was found in plot UB2. Nitidulidae species occur frequently on tree trunks feeding on exuding sap. Adult *Cryptarcha strigata* inhabit the *Quercus robur* trunks, near sap runs, in which the larval stages develop. They rarely occur on exuding sap of *Populus tremula* trunks^68^. Adult and larval *Glischrochilus hortensis* inhabit the fermented sap of *Q. robur* and are found under the bark of fallen and decaying trunks of *B. pendula* and *P. tremula*. In addition, the larvae have also been recorded in fermented berries, vegetables and mushrooms^68,69^. *Glischrochilus grandis* is a common inhabitant of decaying tree sap on *B. pendula* and *Q. robur* in which its larvae develop. It is also known to inhabiting tinder fungi, rotten berries and various other decaying organic matters^70–72^. *Soronia grisea* is confined to oak and mixed (coniferous-deciduous) forests where it frequently occurs on the sap of *Q. robur* and *Salix* sp.^68,72^. Thus, beer traps, which caught most Nitidulidae species, were located on trunks with sap runs. The highest abundance of Nitidulidae species is associated with sites, which had the highest number of trees surviving after the 2010 wildfire. Completely burned sites, where such trees are absent, had the lowest abundance of Nitidulidae specimens caught in beer traps.

In plots LS1 and UB2, the number of Scarabaeidae specimens was higher than in other study plots. *Protaetia marmorata* is one of the most common species caught by beer traps. This species inhabits mixed forests, deciduous forests, pine forests, alleys, public parks and shelterbelts^73–76^. Oleksa et al.^77^ showed that this species has no specific preference for any deciduous tree species. Their larvae develop in holes in dead deciduous trees taking three years to develop^73,75^. They primarily develop in *Q. robur*^78^, confirmed by Ruchin et al.^76^. In such sites, *Protaetia marmorata* was caught in greatest numbers in the beer traps, while the catch was extremely low in sites where *Q. robur* was absent.

The rare European species, *Protaetia fieberi*, has been found in all studied sites. Previously, Ruchin et al.^79^ noted that this species is regularly observed in various habitats in central European Russia. Like other Scarabaeidae species, the highest abundance of *Protaetia fieberi* specimens was found in plot LS1. Its larvae are saproxylophagous inhabiting tree holes in *Quercus*, *Tilia*, *Fagus*, *Salix*, *Populus*, made by various species of woodpeckers, owls and small mammals^80^.

The greatest number of the Cerambycidae species was found in plot SS2. Its value was twice lower in plots UB2 and CF1. All of the Cerambycidae species recorded are saproxylic. *Leptura thoracica*, *L. quadrifasciata*, *Rhagium mordax*, *Stenocorus meridianus* and *Obrium cantharinum* dominated in number of specimens. *Leptura thoracica* and *L. quadrifasciata* were found in all of the studied habitats. *Leptura quadrifasciata* larvae develop in dead or rotting wood, especially in the lower trunk of standing trees, tree stumps, felled trunks and branches. A wide range of the host trees was recorded, including alder, aspen, beech, birch hazel, oak, poplar, sallow, willow, elder but with birch apparently preferred. The larvae live in the moist or dry wood^81^. It is well known that *Leptura thoracica* is a polyphagous species associated with deciduous trees (e.g. *Populus*, *Betula*, *Tilia*, *Salix*, *Fagus*). Its larvae inhabit the dead, rotten wood of large trunks^82–84^. Previously, Danilevsky et al.^85^ found that this species is most often collected in sites where *Betula* species dominate. It is considered that the high number of the adult *Leptura quadrifasciata* and *Leptura thoracica* could be explained by a high number of fallen and rotten birch trunks in the fire-damaged habitats.

*Rhagium mordax* is one of the most common species in the Republic of Mordovia^86,87^. Nevertheless, it has been found only in seven study plots, with the highest number of specimens in the control (unburned) plots UB1 and UB2. Its larvae develop under bark of dead coniferous and deciduous trees^88^. However, in many study plots, we found fallen trunks without bark, which could limit the amount of microhabitat available for *Rhagium mordax* larvae. Conversely, in control plots (UB1 and UB2), there were enough fallen trunks with bark still attached which probably led to the higher abundance of this species in the unburned sites. *Stenocorus meridianus* larvae develop in the roots of dead deciduous trees^88^. This species was characterised by the high abundance in plot SS2 where the number of specimens of *Leptura thoracica* and *Leptura quadrifasciata* was also quite high. *Obrium cantharinum* larvae take 1–2 years to develop in or under very dry bark of dead branches and stems of aspen. This species prefers *P. tremula* trunks of 15–20 cm width with thin bark and in sunny conditions^89,90^. Among all the studied sites, *Obrium cantharinum* occurred in the highest number in plot LS1. This was the only site where were many *P. tremula* trunks were to be found.

The regular observations of the Cerambycidae species in the study area could be due to the fact that they are anthophilous species^91^. Such species as *Leptura quadrifasciata*, *Stenocorus meridianus*, *Rhagium mordax* visit flowering plants in the undergrowth layer in small well-illuminated sites in the burned areas. Some of the anthophilous beetle species occur regularly in sites damaged by the surface fires^92^. In such post-fire conditions, we observe the active development of the herb layer and a well habitat illumination. As a result, in such habitats, the flowering plants serve as a feeding source for anthophilous beetles^93^. In addition, these species are able to actively fly and move easily into the study sites from the adjacent unburned areas.

Some authors have previously observed a greater abundance of Lepidoptera in burned areas than in unburned ones^94–96^. Recovery of butterfly populations is observed at different times after fires^94,97^. In the Mediterranean forests, in the year after a crown fire, there was a tendency for an increase in the number of butterflies closer to the epicenter of the fire^37^. After a fire, Lepidoptera seem to be attracted to "patches" of grassy vegetation and forest clearings because of their habitat preferences^98^. In addition, Grundel et al.^94^ found that stand heterogeneity is necessary to maintain a complex forest canopy structure that supports lepidopteran reproduction. Healthy trees and undergrowth vegetation provide an ideal habitat for preserving food for Lepidoptera species and provides protection from predators. The results of our research show that the number of butterflies decreases in areas of crown fires. In such conditions, there is no undergrowth or well-defined herb layer. In this case, the lack of options for reproduction and nutrition has a negative effect on this group of invertebrates. It is possible that the availability of Lepidoptera for various insectivorous birds and mammals is also increasing in this case. The highest numbers of Lepidoptera were recorded in the control areas and plots LS1, where a low-intensity surface fire had occurred. All these sites have all the necessary conditions and resources for the life cycle of Lepidoptera.

In our study, Vespidae were the most numerous family among the Hymenoptera. The dominant species from this order were *Vespa crabro*, *Vespula vulgaris*, *Dolichovespula media*, and *Vespula germanica*. Their total number reached 96.6%, while the rest of the species were only represented by single specimens. These are characteristic species for central European Russia^99^. Some authors have also noted the abundance of specimens of this family in traps with beer^100,101^. These wasps are quite demanding on their habitat, for feeding and nesting they require open, sunny areas, characterised by the presence of a variety of flora, shelter, and hunting opportunities. The activity of the species is influenced by illumination, ambient temperature, and humidity^102–104^. Clemente et al.^105^ determined that an improvement in food availability may be beneficial for social wasps and will maintain a population even after a fire. By exploiting a range of environmental factors like sources of water, vegetable fiber, nectar and prey, social wasps display an opportunistic character. They return to places with a large supply of resources or food in search of feed optimisation and reduced search effort^106^.

According to our observations, if the sample plot did not have time to regrow with secondary vegetation, then it is not attractive for Vespidae. This is confirmed by the data on the number of wasps on plots CF1, CF2, and SS2, where the secondary layer of vegetation (shrubs and tree undergrowth) is not developed. When grassy vegetation appears, nectar plants with open nectaries are found, these are ideal food sources due to the wasps' short mouthparts. This is especially true for *D. media*, *Dolichovespula saxonica* and *V. vulgaris*. *V. crabro*, as a rule does not visit such inflorescences to collect nectar but to hunt flies and bees feeding on flowers. When young trees appear, especially birch trees, *Vespa crabro* workers can visit them en masse, gnawing the young bark to make the shells of their nests. This was observed especially well on the outskirts of burnt forests (plots SS3 and SS4). The dry wood of dead trees is a source of pulp (chewed vegetable fiber), from which wasps make their nests^107,108^. However, wasps are attracted not by burnt trees, but by broken trunks and stumps with bare wood. *V. crabro* and *V. vulgaris* prefer to collect pulp on rotten birch trunks and stumps. *D. media* and *D. saxonica* are less demanding on tree species and can collect pulp even from dry coniferous trunks. In addition, the presence of shrub vegetation is a positive factor, since many wasps hunt on the leaves of shrubs. *D. media* and *D. saxonica*, place their nests freely, fixing them on the branches of trees and bushes at a height of 0.5–5 m, sometimes higher. *V. crabro* prefer to occupy hollows in trunks, and *V. vulgaris—*abandoned underground rodent burrows. Thus, the combination of conditions for the construction of nests, the possibilities of hunting and the consumption of nectar, determines the development of Vespidae in burnt forests. In the 9th year after the fire, the conditions of the areas where there were upper and lower fires of high intensity had not yet had time to form in an optimal combination. Therefore, in these areas, the number of individuals of Vespidae was lower than in other areas.

The most significant number of Anisopodidae individuals was found in plot UB3. *Sylvicola punctatus* was the dominant species (88.7% of the total number of Anisopodidae specimens), which was found in all areas except plot CF1. It is the most frequently encountered species in the pine and deciduous forests of the Mordovia State Nature Reserve^109^. Larvae of this species live in rotting plant materials (fungi, leaves, manure, decaying wood), fermenting sap^110^. The family Pallopteridae was represented by three species in small numbers. *Toxoneura saltuum* and *Toxoneura trimacula* larvae develop in the stems of *Angelica sylvestris* L. and *Heracleum sibiricum* L.^111^.

The greatest number of Ulidiidae specimens was recorded on plot LS1. Two species of the family Ulidiidae have been found, one of which, *Pseudotephritis millepunctata*, was discovered for the first time in Europe^112^. Larvae of this species are common inhabitants of weakened and dying deciduous trees such as *Alnus hirsuta* and *Quercus mongolica*^113^. Apparently, in Europe, they also prefer similar species from the genera *Alnus* and *Quercus*. This is indirectly confirmed by the fact that the greatest number of *Pseudotephritis millepunctata* is on plot LS1, where, unlike other sites, there were many *Alnus glutinosa* trees weakened after the wildfire.

Plot LS1 and plot UB1 had the largest number of Drosophilidae individuals with *Drosophila obscura* being the most frequently found species, followed by the closely related *Drosophila bifasciata* both of which belong to the *D. obscura* species group. Both are widely distributed throughout Europe in the forest zone^114–116^. In this study, we found a fairly uniform distribution of *D. obscura* and *D. bifasciata* in areas with high and low intensity fires, on the outskirts of the fire and in control. Both of these species belong to the ecological group of xylosaprobionts, whose larvae develop under the bark of trees, in wet tissues, and in the sap runs^115,117^. *Amiota semivirgo* was also almost uniformly distributed with a slight increase in numbers in areas with moderate fire intensity (plot SS3 and plot SS1). Their larvae are also known to develop under the bark of trees^118^. *Gitona distigma* was found in all areas with maximum numbers recorded in plot SS3; its larvae develop in the inflorescences of plants of the family Asteraceae^117^.

*Leucophenga quinquemaculata* is the only species from the ecological group of mycetobionts with a very uniform distribution over all plots. The larvae of this fly develop in the fruiting bodies of various tinder fungi, especially *Piptoporus betulinus* and *Fomitopsis pinicola*^119,120^.

Three species of Drosophilidae (*Amiota alboguttata*, *Amiota rufescens*, and *Scaptodrosophila rufifrons*) were not collected in single numbers and did not show a uniform distribution. Both *Amiota* species were most common in low-intensity fire areas (plot LS1). Larvae of *A. alboguttata* were previously recorded under oak bark and it has also been bred from the fungus *Daldinia concentrica*, which grew on a burnt birch^121^. This fungus is a saprotrophic species living on dead and rotting wood. The biology of *A. rufescens* remains unknown but given the apparent similarity of the distribution of these flies with that of *A. alboguttata* it can be assumed that the larval development sites may be similar.

The largest number of Lonchaeidae was recorded on plot LS1. *Lonchaea carpathica* and *Lonchaea limatula* being dominant ands representing 97.5% of the total. Both species predominate in deciduous forests; larvae develop in decaying tree trunks, more often in birch^122–125^. In areas dominated by these species, there was a significant amount of wood fall. On plot LS1, there was the maximum number of all Lonchaeidae individuals (59.1%) with the maximum species diversity (7 out of 8 species). This is because the larvae of all Lonchaeidae species are saproxylic and prefer deciduous trees with bark still attached. This area is home to different types of deciduous trees and has many fallen trunks (*Betula, Populus, Alnus, Quercus, Ulmus*), which are used by the larvae of several different species.

The family Muscidae was most common in plots LS1 and UB1, and was represented by 10 species. *Phaonia pallida* (61.0% of the total number of specimens in the family) was particularly significant. The larvae of this species live in rotting plant remains, dead and rotten wood with the adult preferring grassy vegetation^126,127^. *P. pallida* occurred in low numbers in the areas of crown fires—plots CF1 and CF2. The number of species from the Pollenidae family was higher on plot UB3. There is little data on the adult biology of this group but there is evidence that their larvae develop in earthworms^128^.

The proportion of other orders (Dictyoptera, Heteroptera, Dermaptera, Mecoptera, Trichoptera) was insignificant in the general species list. Those species fall into crown traps irregularly. However, it should be noted that, for example, *Panorpa* sp. was found only in control areas, and species of the order Heteroptera were more common in burnt areas.

In natural ecosystems, various catastrophic impacts on the insect fauna could lead to the similar consequences. For instance, windblow impact assessments following storm Vivian in Germany showed a considerable increase in species diversity and abundance of insects^129^. A similar effect was found after wildfires in Russia^22^, Italy, Czechia^38,130^ for many insect groups. In the disturbed forests, a greater amount of the sunlight reaches the woodland floor, which in turn allows herbs, shrubs and young trees to developed, which are preferred by phytophagous, anthophyllous and some other insect groups. In their turn, the latter insects are the prey for predatory insects (e.g. Neuroptera, Raphidioptera). In the open gaps in the forest, the sunlight favours their reproduction and larval development on herbs and shrubs, as well as on forest edges^92,131,132^. Usually, such effects, which cause an increase in the species diversity and biomass, are a pattern associated with after affects of low intensity wildfire.

Conversely, after wildfire of high intensity (e.g. crown fire and partially severe surface fire) it takes a long time for the complete insect fauna to be restored. The crown fire damages the ecosystem in all levels, when plants of all forest layers are being died. In such sites of the study area, we found the lower species diversity, with the small number of the caught specimens. If such wildfire damages a small site, its further restoration is supported by the insect penetration from the adjacent unburned areas^133,134^. Sometimes, such sites damaged by the crown fire are rounded by the areas damaged by the surface fire. In this case, the insect fauna restoration will take a long time on sites damaged by the crown fire.

Noteworthy, after the wildfire there was a high number of the fallen trunks in many of the study sites. This high amount of decaying timber attracts many saproxylic invertebrates^92,93,135,136^. This especially true of dying and fallen deciduous trees such as *Populus*, which can support large numbers of saproxylic insect larvae. In plot LS1 and some other study sites, the large amount of dead wood caused a considerable increase in the number and diversity of saproxylic species present. The *Rh. mordax* abundance indicates the relation between dead wood availability and species numbers, its larvae develop under bark of both coniferous and deciduous trees. However, in study plots damaged by the highly intensive fire, the bark was damaged and missing and as a result, the number of *Rh. mordax* specimens was extremely low.

We can conclude that in some areas damaged by wildfire of 2010, the insect fauna has been restored. This is indicated by the high species composition similarity of the fire-damaged plot LS1 and the control (unburned) plot UB3. Both sites are located nearby the small rivers and do have a quite similar soil moisture and vegetation character. Plots SS1 and SS2 are also quite similar to the control (unburned) sites in terms of the species diversity (Fig. 4). Therefore, in sites damaged by the low vigorous surface fire in 2010, the insect species diversity is quite similar to one in the unburned areas by the ninth post-fire year.

## Conclusions

The fire-damaged study sites showed differences in terms of the species diversity and number of the specimens caught using crown bait traps. Sites damaged by a crown fire in 2010 do now considerably differ from all other study plots in terms of both species diversity and overall number of specimens caught. This clearly indicates the catastrophic character of crown fires and indicates the long period required for full insect fauna restoration. In the study plots damaged by low intensity surface fires, we found insect fauna restoration by the ninth post-fire year. By that time, these low intensity fire sites differed little in species diversity and number of the specimens from the control (unburned) study plots. The edges of the burned areas also differ from other study sites in the insect species diversity, with a high similarity to each other.

Trends in the post-fire fauna restoration have been revealed for several insect groups. Increased distance from the burned area edge promoted the increase in the number of caught specimens of Scarabaeidae, Nitidulidae, Muscidae, Ulidiidae, and Vespidae and the decrease in the number of specimens of Platystomatidae and Cerambycidae. Nevertheless, all insect groups were characterised by the decline in the species diversity and the number of the specimens in sites damaged by the crown fire.

Our results demonstrate that the presence of dead trunks acts to maintain the abundance of some insect groups and certain insect populations. Perhaps, the presence of the dead wood (especially, deciduous trees) leads to the population restoration of some insect taxa.

## Supplementary Information

Supplementary Information 1.

Supplementary Information 2.

## Data Availability

Applicable.
