# Supplementary material for: Post-fire insect fauna explored by crown fermental traps in forests of the European Russia
Source: Sci Rep. 2021 Oct 29;11:21334. doi: 10.1038/s41598-021-00816-3 (PMC8556309; doi:10.1038/s41598-021-00816-3)
Supplement: Supplementary file 1 — Supplementary Information 1. [file 41598_2021_816_MOESM1_ESM.doc]

**Appendix A.** Description of habitats

Plot SS1: square 275 (Moderately vigorous surface fire).

Canopy layer is scarce, consisting of *Pinus sylvestris* (80%), *Betula pendula* (20%). Understory layer is represented by scarce trees of *Betula pendula*. Shrub layer is almost absent (only solitary individuals of *Salix caprea*), with seedlings of *Betula pendula*, *Populus tremula* and *Pinus sylvestris.* Herb layer consists of *Calamagrostis epigejos*, *Melampyrum pratense*, *Festuca pratensis*, *Solidago virgaurea*, *Pteridium aquilinum*, *Hieracium umbellatum*, *Calamagrostis* *arundinacea*, *Convallaria majalis*, *Rubus saxatilis* and *Vaccinium vitis-idaea*. The fallen deadwood consists of a few *B. pendula* and *P. sylvestris* trees.

Plot LS1: square 299 (Low vigorous surface fire).

The canopy layer is scarce consisting of *Betula pendula* (100%). The understory layer consists of *Betula pendula, Populus tremula* and *Tilia cordata*. Shrub layer is dense, with cover of up to 80%. It includes *Acer platanoides, Sorbus aucuparia, Prunus padus, Frangula alnus, Euonymus verrucosus, Rubus idaeus, Salix caprea, Lonicera xylosteum*and seedlings of of *Quercus robur*. Herb layer is represented by *Calamagrostis epigejos, Melampyrum pratense, Convallaria majalis, Veronica chamaedrys, Lathyrus vernus, Stellaria holostea, Pulmonaria obscura, Poa nemoralis, Rubus saxatilis, Melica nutans, Fragaria vesca, Pimpinella saxifraga, Tanacetum vulgare, Epilobium angustifolium, Carex digitata, Aegopodium podagraria, Maianthemum bifolium, Festuca gigantean, Calamagrostis arundinacea, Dryopteris cristata, Galium mollugo* and *Erigeron annuus*. The fallen deadwood consists of a considerable number of dead or dying trees of *B. pendula*, *Populus tremula*, *Alnus glutinosa*, and a few *Quercus robur*

Plot CF1: square 302 (Active crown fire).

The canopy layer is absent due to the complete destruction by the crown fire in 2010. Woodland vegetation recovery is mainly due to regenerating Betula pendula. There is a scarce undergrowth of *Betula pendula* and rarer *Populus tremula*. The shrub layer is represented by solitary individuals of *Chamaecytissus ruthenicus*. Herb layer is represented by *Calamagrostis epigejos, Veronica spicata, Senecio jacobaea, Polygonum dumetorum, Rubus saxatilis, Pulsatilla patens, Solidago virgaurea, Convallaria majalis, Hieracium umbellatum, Viola rupestris, Melampyrum pratense, Polygonatum odoratum, Carex digitata, Pimpinella saxifraga, Jurinea cyanoides, Hypericum perforatum, Achillea millefolium, Campanula rotundifolia, Dracocephalum ryuschianum* and*, Carum carvi*. The fallen deadwood consists of a considerable number of dead or dying trees of *P. sylvestris* and *B. pendula*.

Plot CF2: square 329 (Active crown fire).

The canopy layer is absent due to the complete destruction by the crown fire in 2010. The woodland recovery consists of *Betula pendula* regeneration. The woody vegetation is represented by the *Betula pendula* undergrowth. Shrub layer consists of *Chamaecytissus ruthenicus*, *Malus sylvestris*. Herb layer is represented by *Calamagrostis epigejos, Epilobium angustifolium, Solidago virgaurea, Veronica spicata, Sedum maximum, Polygonatum odoratum, Hypericum perforatum, Senecio jacobaea, Hieracium umbellatum, Pimpinella saxifraga, Viola rupestris, Tanacetum vulgare, Achillea millefoilum, Viola mirabilis, Convallaria majalis, Melampyrum pratense, Carex digitata, Geranium sanguinea, Poa trivialis, Festuca pratensis, Campanula rotundifolia, Erigeron annuus* and*, Pilosella officinarum*. The fallen deadwood consists of a considerable number of dead trees of *Pinus sylvestris* and *Picea abies* and a few *Betula pendula*.

Plot SS2: square 330 (Moderately vigorous surface fire).

Canopy layer consists of Pinus sylvestris (100%). Understory layer was destroyed by wildfire. Shrub layer is represented by *Chamaecytissus ruthenicus, Genista tinctoria, Rosa majalis*, as well as *Betula pendula* and *Populus tremula* which form dense thickets. Herb layer consists of *Epilobium angustifolium, Calamagrostis arundinacea, Rubus saxatilis, Convallaria majalis, Carum carvi, Solidago virgaurea, Hieracium umbellatum, Melampyrum pratense, Hypericum perforatum, Galium* *boreale, Polygonatum odoratum* and *Dracocephalum ryuschianum*. The fallen deadwood consists of a considerable number of dead trees of *P. sylvestris* and *B. pendula*with a few *P. abies*.

Plot SS3: square 358 (edge of burned area damaged by moderately vigorous surface fire).

Canopy layer is absent. Woody vegetation is represented by regenerationl of *Betula pendula* (coverage of 45–55%), *Tilia cordata, Salix caprea* and*, Populus tremula*. Shrub layer includes *Rubus nessensis, Rubus idaeus, Chamaecytissus ruthenicus, Genista tinctoria, Euonymus verrucosus*. Herb layer consists of *Epilobium angustifolium* (40%), *Calamagrostis epigejos, Vicia sylvatica, Rubus saxatilis, Galium boreale, Deschampsia cespitosa, Veronica chamaedrys, Veronica officinalis, Polygonum dumetorum, Melica nutans, Campanula rotundifolia, Filipendula ulmaria, Vicia cracca, Stellaria graminea, Convallaria majalis, Poa nemoralis, Carex digitata, Artemisia vulgaris, Erigeron canaensis* and *Agrostis tenuis*. The fallen deadwood consists of a very large number of dead *P. sylvestris* trees with a few *B. pendula*.

Plot SS4: square 384 (edge of burned area damaged by moderately vigorous surface fire).

The plot is covered by *Betula pendula* regenerationl after the wildfire impact. Shrub layer consists of *Frangula alnus, Sorbus aucuparia, Rubus idaeus, Rubus nessensis, Chamaecytissus ruthenicus*, and seedling *Pinus sylvestris*. Herb layer is represented by *Pteridium aquilinum, Convallaria majalis, Calamagrostis epigejos, Pilosella officinarum, Vaccinium vitis-idaea, Calamagrostis arundinacea, Trientalis europaea, Fragaria vesca, Epilobium angustifolium* and*, Calluna vulgaris*. The fallen deadwood consists of a very large number of dead *P. sylvestris* trees with a few *Betula pendula* and *Populus tremula*.

Plot UB1: 408 (unburned area, coniferous forest).

Canopy layer consists of *Pinus sylvestris* (90%) and *Picea abies* (10%). Understory layer includes *Alnus glutinosa, Betula pendula, Picea abies*. Shrub layer consists of Fr*angula alnus, Sorbus aucuparia, Rubus idaeus, Acer platanoides, Rubus nessensis, Malus sylvestris*, undergrowth of *Quercus robur*, and *Prunus padus*. Herb layer includes *Dryopteris cristata, Rumex acetosella, Luzula pilosa, Poa nemoralis, Melampyrum pratense, Trientalis eauropaea, Stellaria holostea, Vaccinium myrtillus, Oxalis acetosella, Carex digitata, Convallaria majalis, Maianthemum bifolium, Dryopteris filix-mas*. The fallen deadwood consists of a few trunks of *P. sylvestris* and *Betula pendula*.

Plot UB2: square 427 (unburned area, coniferous forest).

Canopy layer consists of *Pinus sylvestris* (100%). Understory layer is formed by *Betula pendula*. Shrub layer consists of *Sorbus aucuparia, Frangula alnus, Salix cinerea, Acer platanoides*, and undergrowth of *Picea abies, Quercus robur*, and *Tilia cordata*. Herb layer is represented by *Calamagrostis canescens, Vaccinium vitis-idaea, Vaccinium myrtillus, Poa nemoralis, Veronica officinalis, Rubus idaeus, Melica nutans, Orthilia secunda, Rubus saxatilis, Convallaria majalis, Melampyrum pratense, Deschampsia cespitosa, Dryopteris carthusiana*. The fallen deadwood consists of a few dead trunks of *P. sylvestris* and *Betula pendula*, with solitary trunks of *Populus tremula* and *Alnus glutinosa*.

Plot UB3: vicinity of the village Rosstanye, 0.8 km of the Protected Area border (unburned area, small-leaved forest).

Canopy layer consists of *Alnus glutinosa* (90%) and *Betula alba* (10%). Understory layer is absent. Shrub layer is formed by *Frangula alnus, Salix cinerea, Salix pentandra*, and undergrowth of *Ulmus laevis, Quercus robur, Pinus sylvestris. Herb layer consists of Humulus lupulus, Tussilago farfara, Calamagrostis epigejos, Rumex confertus, Rumex acetosa, Rubus saxatilis, Tanacetum vulgare, Artemisia vulgare, Polygonum dumetorum, Rubus caesia, Galium mollugo, Festuca pratensis, Phleum pratense, Achillea millefoilum, Carex rostrata, Lysimachia vulgaris, Calamagrostis canescens, Solanum dulcamara*. The fallen deadwood consists of a few dead trunks of *Betula pendula*, *Populus tremula* and *Alnus glutinosa*.

| 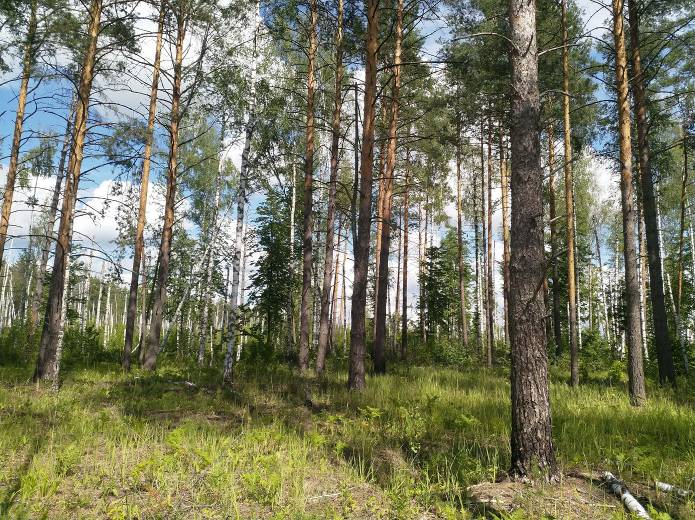 | 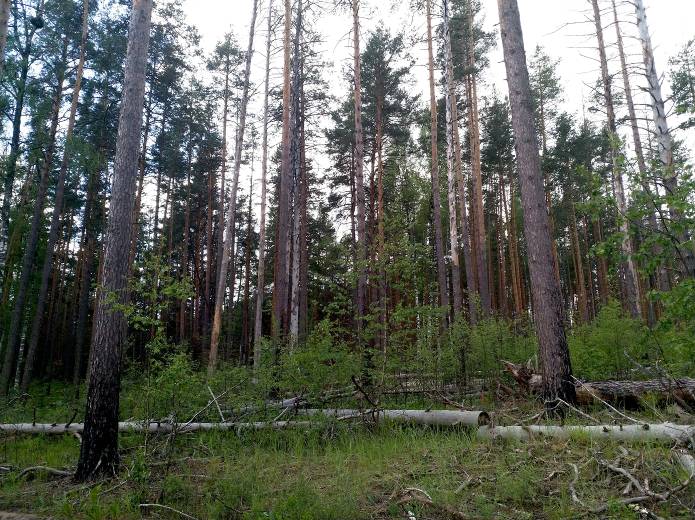 |
| --- | --- |
| A | F |
| 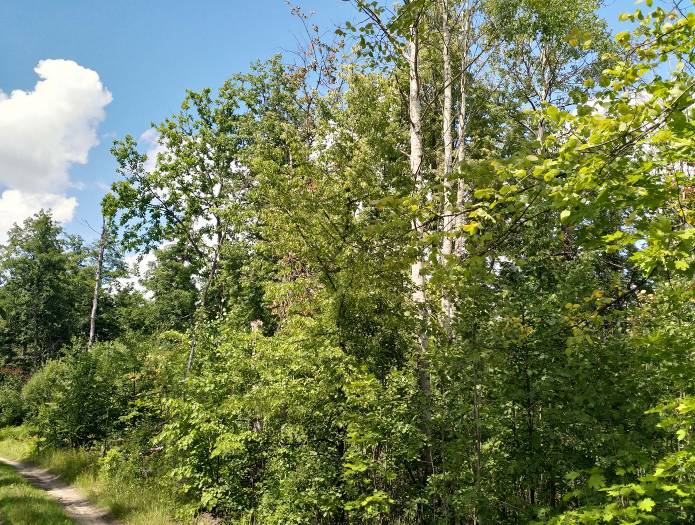 | 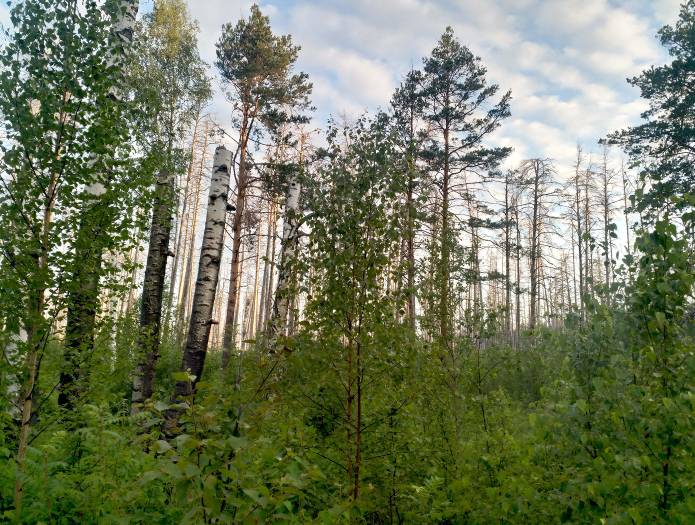 |
| B | G |
| 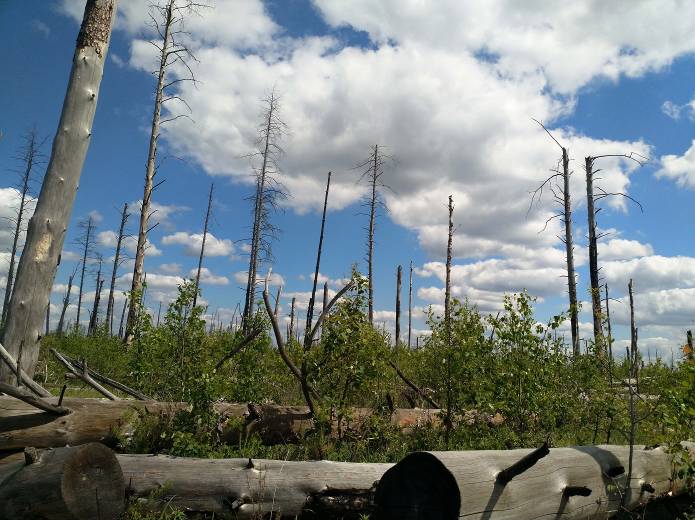 | 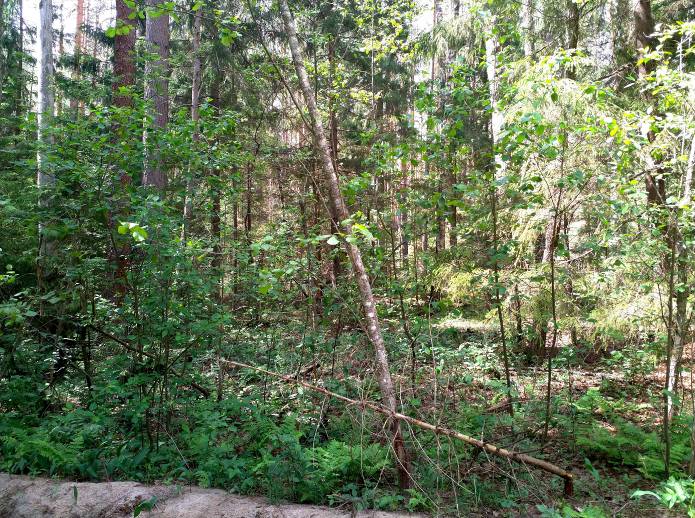 |
| C | H |
| 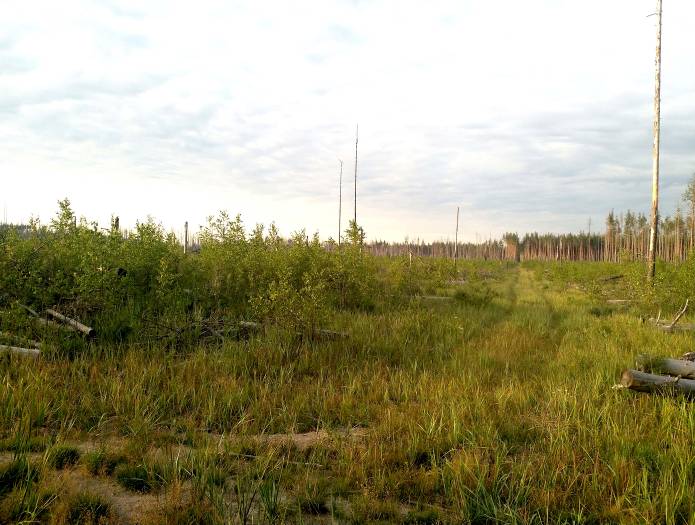 | 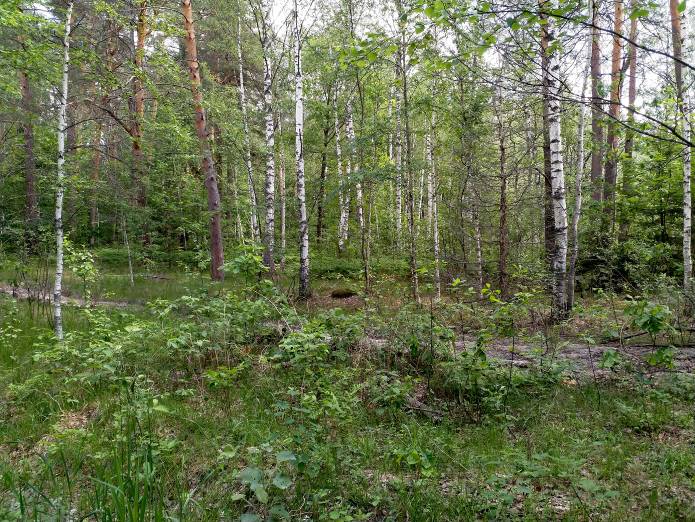 |
| D | I |
| 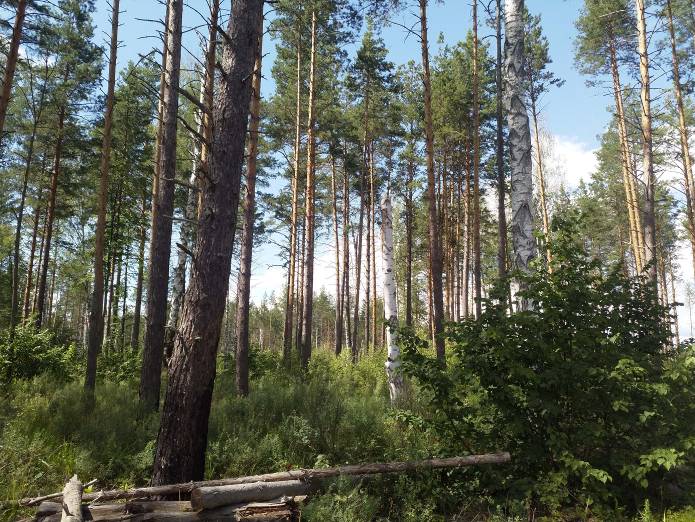 | 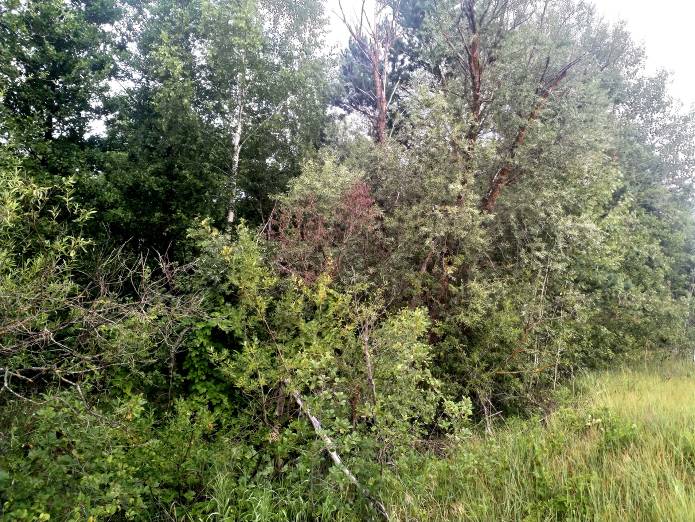 |
| E | J |

Photos of the study plots: A – plot SS1; B – plot LS1; C – plot CF1; D – plot CF2; E – plot SS2; F – plot SS3; G – plot SS4; H – plot UB1; I – plot UB2; J – plot UB3.
