# Supplementary material for: Post-fire insect fauna explored by crown fermental traps in forests of the European Russia
Source: Sci Rep. 2021 Oct 29;11:21334. doi: 10.1038/s41598-021-00816-3 (PMC8556309; doi:10.1038/s41598-021-00816-3)
Supplement: Supplementary file 2 — Supplementary Information 2. [file 41598_2021_816_MOESM2_ESM.doc]

**Appendix B.** The list of insect species found in burned and control plots

| **Taxon** | plot SS1 | plot LS1 | plot CF1 | plot CF2 | plot SS2 | plot SS3 | plot SS4 | plot UB1 | plot UB2 | plot UB3 |
| --- | --- | --- | --- | --- | --- | --- | --- | --- | --- | --- |
| **DERMAPTERA** |  |  |  |  |  |  |  |  |  |  |
| **Forficulidae** |  |  |  |  |  |  |  |  |  |  |
| *Forficula auricularia* Linnaeus, 1758 |  |  |  |  |  |  |  |  |  | 1 |
| **DICTYOPTERA** |  |  |  |  |  |  |  |  |  |  |
| **Ectobiidae** |  |  |  |  |  |  |  |  |  |  |
| *Ectobius lapponicus* (Linnaeus, 1758) |  |  |  |  | 1 |  |  |  |  | 3 |
| **HETEROPTERA** |  |  |  |  |  |  |  |  |  |  |
| **Miridae** |  |  |  |  |  |  |  |  |  |  |
| *Deraeocoris ruber* (Linnaeus, 1758) |  | 1 |  | 1 |  |  |  |  |  |  |
| *Polymerus unifasciatus* (Fabricius, 1794) |  |  |  |  |  |  |  |  | 2 | 1 |
| **Pentatomidae** |  |  |  |  |  |  |  |  |  |  |
| *Dolycoris baccarum* (Linnaeus, 1758) |  |  |  |  |  |  | 1 |  |  |  |
| *Palomena prasina* (Linnaeus, 1760) |  |  | 2 |  | 1 |  | 2 |  |  |  |
| **RAPHIDIOPTERA** |  |  |  |  |  |  |  |  |  |  |
| **Raphidiidae** |  |  |  |  |  |  |  |  |  |  |
| *Dichostigma flavipes* (Stein, 1863) |  | 1 | 1 |  | 2 |  |  |  | 8 |  |
| **NEUROPTERA** |  |  |  |  |  |  |  |  |  |  |
| **Chrysopidae** |  |  |  |  |  |  |  |  |  |  |
| *Chrysopa gibeauxi* (Leraut, 1989) |  |  | 1 |  | 1 |  |  | 1 |  |  |
| *Chrysoperla carnea* (Stephens, 1836) |  | 1 | 18 | 6 |  | 1 | 1 |  |  | 1 |
| *Chrysotropia ciliata* (Wesmael, 1841) |  | 3 |  |  | 2 | 1 |  | 4 | 34 | 29 |
| *Cunctochrysa albolineata* (Killington, 1935) |  |  |  | 1 |  |  |  |  |  |  |
| *Nineta alpicola* Kuwayama, 1956 | 1 | 26 | 1 |  | 6 |  | 1 | 2 | 6 | 20 |
| *Nineta flava* (Scopoli, 1763) |  | 1 |  |  |  |  |  |  |  |  |
| *Nineta vittata* (Wesmael, 1841) |  | 7 |  |  |  |  |  | 1 | 2 |  |
| *Nothochrysa fulviceps* (Stephens, 1836) |  |  |  |  |  |  |  |  |  | 1 |
| *Pseudomalada flavifrons* (Brauer, 1851) | 1 | 2 |  |  | 6 | 4 | 1 |  |  | 2 |
| *Pseudomalada prasinus* (Burmeister, 1839) | 9 | 80 | 12 | 9 | 64 | 35 | 23 | 3 | 22 | 18 |
| *Pseudomalada ventralis* (Burmeister, 1839) |  | 4 |  |  | 1 |  | 3 | 1 | 2 | 3 |
| **COLEOPTERA** |  |  |  |  |  |  |  |  |  |  |
| **Dytiscidae** |  |  |  |  |  |  |  |  |  |  |
| *Ilybius fuliginosus* (Fabricius, 1792) |  |  |  |  |  |  |  | 1 |  |  |
| **Histeridae** |  |  |  |  |  |  |  |  |  |  |
| *Gnathoncus buyssoni* Auzat, 1917 |  |  |  |  | 1 |  |  |  |  |  |
| *Platysoma elongatum* (Thunberg, 1787) |  |  |  |  | 5 | 2 |  |  |  |  |
| *Platysoma lineare* Erichson, 1834 |  |  |  |  |  | 1 | 1 |  |  |  |
| **Silphidae** |  |  |  |  |  |  |  |  |  |  |
| *Oiceoptoma thoracicum* (Linnaeus, 1758) |  |  |  |  |  |  |  |  |  | 1 |
| **Staphylinidae** |  |  |  |  |  |  |  |  |  |  |
| Staphylinidae sp. | 1 |  |  |  | 1 | 7 | 7 | 3 |  | 1 |
| *Quedius dilatatus* (Fabricius, 1787) | 1 | 1 |  |  | 2 |  | 1 | 9 | 1 | 5 |
| **Scarabaeidae** |  |  |  |  |  |  |  |  |  |  |
| *Cetonia aurata* (Linnaeus, 1758) |  | 2 | 2 | 1 | 1 | 3 | 7 |  |  | 2 |
| *Gnorimus variabilis* (Linnaeus, 1758) |  | 3 |  |  |  |  |  | 1 | 2 |  |
| *Protaetia fieberi* (Kraatz, 1880) | 4 | 15 | 3 | 4 | 2 | 2 | 7 | 1 | 1 | 3 |
| *Protaetia marmorata* (Fabricus, 1792) | 3 | 44 |  | 1 | 3 | 2 | 17 | 29 | 65 | 13 |
| *Protaetia speciosissima* (Scopoli, 1786) |  | 2 |  |  |  |  |  |  |  |  |
| *Protaetia cuprea volhyniensis* (Gory & Percheron, 1833) |  | 6 |  |  |  |  | 4 | 7 | 1 | 5 |
| *Trichius fasciatus* (Linnaeus, 1758) |  |  |  |  |  |  |  | 2 |  |  |
| **Scirtidae** |  |  |  |  |  |  |  |  |  |  |
| *Contacyphon* sp. |  |  | 1 |  |  |  |  |  |  |  |
| *Microcara testacea* (Linnaeus,1767) |  |  |  |  |  |  |  | 1 |  |  |
| **Eucnemidae** |  |  |  |  |  |  |  |  |  |  |
| *Otho sphondyloides* (Germar, 1818) |  |  |  |  |  | 1 |  |  |  |  |
| **Elateridae** |  |  |  |  |  |  |  |  |  |  |
| *Agrypnus murinus* (Linnaeus, 1758) |  |  | 1 |  |  |  |  |  |  | 2 |
| *Ampedus balteatus* (Linnaeus, 1758) |  |  |  | 1 |  |  |  |  |  |  |
| *Ampedus cinnabarinus* (Eschscholtz, 1829) |  | 1 | 5 |  |  | 3 |  |  |  |  |
| *Ampedus elongatulus* (Fabricius, 1787) |  | 2 |  |  |  |  |  | 1 |  |  |
| *Ampedus pomorum* (Herbst, 1784) |  |  |  |  | 1 |  |  |  |  |  |
| *Ampedus praeustus* (Fabricius, 1792) |  |  |  |  |  |  |  |  | 1 |  |
| *Ampedus sanguinolentus* (Schrank, 1776) |  |  |  |  |  |  |  |  |  | 1 |
| *Ectinus aterrimus* (Linnaeus, 1760) |  | 1 |  |  |  |  |  |  |  |  |
| *Elater ferrugineus* Linnaeus, 1758 | 1 | 2 |  |  |  |  |  |  |  | 1 |
| *Melanotus castanipes* (Paykull, 1800) |  |  |  |  | 1 | 2 |  |  |  |  |
| *Prosternon tesselatum* (Linnaeus, 1758) |  |  |  |  | 1 |  |  |  | 1 | 2 |
| **Buprestidae** |  |  |  |  |  |  |  |  |  |  |
| *Buprestis haemorrhoidalis* Herbst, 1780 |  |  |  |  |  | 1 |  |  |  |  |
| *Phaenops cyanea* (Fabricius, 1775) |  |  |  |  |  | 1 |  |  |  |  |
| **Lycidae** |  |  |  |  |  |  |  |  |  |  |
| *Lygistopterus sanguineus* (Linnaeus, 1758) |  |  |  |  | 1 | 2 | 5 |  |  |  |
| **Cantharidae** |  |  |  |  |  |  |  |  |  |  |
| *Cantharis livida* Linnaeus, 1758 |  | 1 |  |  |  |  |  |  |  |  |
| *Cantharis pellucida* Fabricius, 1792 |  |  |  |  |  | 1 |  |  |  |  |
| *Cantharis rufa* Linnaeus, 1758 |  |  |  |  |  | 1 |  |  |  | 1 |
| **Dermestidae** |  |  |  |  |  |  |  |  |  |  |
| *Attagenus schaefferi* (Herbst, 1792) |  |  |  |  |  | 1 | 22 | 1 | 8 |  |
| *Globicornis emarginata* (Gyllenhal, 1808) |  |  |  |  |  |  |  |  | 7 |  |
| *Megatoma undata* (Linnaeus, 1758) |  |  |  |  |  |  |  |  | 1 |  |
| *Trogoderma glabrum* (Herbst, 1783) | 1 | 1 |  |  |  | 1 | 59 | 3 | 9 | 1 |
| **Ptinidae** |  |  |  |  |  |  |  |  |  |  |
| *Dorcatoma robusta* A. Strand, 1938 | 1 |  |  |  |  |  | 2 |  |  |  |
| *Dorcatoma dresdensis* Herbst, 1792 |  |  |  |  |  |  | 1 |  |  |  |
| **Throscidae** |  |  |  |  |  |  |  |  |  |  |
| *Trixagus* sp. |  |  |  |  |  |  | 1 |  |  | 2 |
| **Cleridae** |  |  |  |  |  |  |  |  |  |  |
| *Allonyx quadrimaculatus* (Schaller, 1783) |  |  |  | 1 |  | 1 |  |  |  |  |
| *Thanasimus femoralis* (Zetterstedt, 1828) |  |  |  |  |  |  |  |  | 1 |  |
| *Trichodes apiarius* (Linnaeus, 1758) |  |  |  | 1 |  |  |  |  |  |  |
| **Melyridae** |  |  |  |  |  |  |  |  |  |  |
| *Dasytes niger* (Linnaeus, 1760) | 1 |  |  |  |  |  |  |  | 1 |  |
| **Nitidulidae** |  |  |  |  |  |  |  |  |  |  |
| *Carpophilus hemipterus* (Linnaeus, 1758) | 3 |  | 3 | 1 |  |  | 1 |  |  |  |
| *Carpophilus* sp. |  |  |  |  |  |  | 1 |  |  |  |
| *Cryptarcha strigata* (Fabricius, 1787) | 6 |  | 1 |  | 2 | 1 | 10 | 5 | 48 | 9 |
| *Cryptarcha undata* (G.-A. Olivier, 1790) |  |  |  |  |  |  |  |  | 1 |  |
| *Cychramus luteus* (Fabricius, 1787) | 1 |  |  |  |  |  |  |  | 4 |  |
| *Cychramus variegatus* (Herbst, 1792) |  |  |  |  |  |  |  |  | 4 |  |
| *Epuraea* sp. | 7 | 1 |  | 2 | 3 |  | 9 |  | 3 | 2 |
| *Glischrochilus hortensis* (Geoffroy, 1785) | 1 |  |  |  | 3 |  | 3 | 6 | 2 | 3 |
| *Glischrochilus grandis* (Tournier, 1872) | 1 |  |  |  | 2 | 1 |  | 2 |  |  |
| *Soronia grisea* (Linnaeus, 1758) |  |  |  |  |  | 2 |  |  | 2 | 6 |
| **Cucujidae** |  |  |  |  |  |  |  |  |  |  |
| *Pediacus depressus* (Herbst, 1797) | 1 |  | 1 |  | 1 |  | 1 |  |  | 5 |
| **Coccinellidae** |  |  |  |  |  |  |  |  |  |  |
| *Calvia decemguttata* (Linnaeus, 1767) |  |  |  |  |  |  |  | 1 |  |  |
| *Calvia quatuordecimguttata* (Linnaeus, 1758) |  |  |  |  |  |  |  |  |  | 1 |
| *Harmonia quadripunctata* (Pontoppidan, 1763) |  | 1 | 1 |  |  |  |  |  |  |  |
| *Sospita vigintiguttata* (Linnaeus, 1758) |  | 1 |  |  |  |  |  |  |  |  |
| **Mordellidae** |  |  |  |  |  |  |  |  |  |  |
| *Tomoxia bucephala* A. Costa, 1854 |  |  |  |  |  |  | 2 |  |  |  |
| **Tenebrionidae** |  |  |  |  |  |  |  |  |  |  |
| *Lagria hirta* (Linnaeus, 1758) | 2 | 2 | 1 |  | 2 | 1 | 3 | 1 | 1 | 4 |
| *Mycetochara axillaris* (Paykull, 1799) | 1 |  |  |  |  |  |  |  |  |  |
| *Upis ceramboides* (Linnaeus, 1758) |  |  |  |  |  | 3 | 1 |  |  |  |
| **Scraptiidae** |  |  |  |  |  |  |  |  |  |  |
| *Anaspis frontalis* (Linnaeus, 1758) |  |  |  |  |  |  | 1 |  |  |  |
| **Cerambycidae** |  |  |  |  |  |  |  |  |  |  |
| *Anastrangalia reyi* (L. Heyden, 1889) |  |  |  |  |  |  | 1 |  |  |  |
| *Arhopalus rusticus* (Linnaeus, 1758) |  |  | 1 | 3 |  | 1 |  |  |  |  |
| *Aromia moschata* (Linnaeus, 1758) |  | 2 |  | 1 |  | 1 | 2 |  |  | 2 |
| *Etorofus pubescens* (Fabricius, 1787) |  |  |  |  |  | 1 |  |  |  |  |
| *Leptura aurulenta* Fabricius, 1793 |  | 1 |  |  |  |  |  |  |  |  |
| *Leptura quadrifasciata* Linnaeus, 1758 | 23 | 15 | 35 | 5 | 35 | 59 | 4 | 18 | 30 | 20 |
| *Leptura thoracica* Creutzer, 1799 | 2 | 13 | 15 | 8 | 94 | 81 | 20 | 49 | 30 | 8 |
| *Lepturalia nigripes* (De Geer, 1775) |  |  | 10 |  |  | 1 |  |  |  | 1 |
| *Necydalis major* Linnaeus, 1758 |  | 1 |  |  | 4 | 2 |  | 3 | 3 | 2 |
| *Obrium cantharinum* (Linnaeus, 1767) | 7 | 18 | 7 | 1 | 4 |  |  |  |  | 1 |
| *Pachyta quadrimaculata* (Linnaeus, 1758) |  |  |  |  | 2 | 1 |  | 1 |  |  |
| *Purpuricenus globulicollis* Dejean, 1839 |  |  |  |  | 1 |  |  |  |  |  |
| *Purpuricenus kaehleri* (Linnaeus, 1758) |  | 1 |  |  |  |  |  |  |  | 1 |
| *Rhagium inquisitor* (Linnaeus, 1758) |  |  |  |  | 2 | 2 |  |  | 4 |  |
| *Rhagium mordax* (De Geer, 1775) | 3 | 3 |  |  | 6 | 4 |  | 41 | 11 | 3 |
| *Rutpela maculata* (Poda von Neuhaus, 1761) |  |  |  |  | 2 |  |  |  |  |  |
| *Spondylis buprestoides* (Linnaeus, 1758) |  |  |  |  | 2 |  |  |  |  |  |
| *Stenocorus meridianus* (Linnaeus, 1758) | 1 |  | 3 | 1 | 18 | 7 |  |  | 3 | 10 |
| *Stictoleptura rubra* (Linnaeus, 1758) |  |  |  |  |  |  |  |  | 1 |  |
| *Strangalia attenuata* (Linnaeus, 1758) |  |  | 1 |  |  | 1 |  |  |  |  |
| *Xylotrechus antilope* (Schoenherr, 1817) |  | 1 |  |  |  |  |  |  |  |  |
| *Xylotrechus rusticus* (Linnaeus, 1758) |  |  |  |  |  |  |  |  | 1 |  |
| **Chrysomelidae** |  |  |  |  |  |  |  |  |  |  |
| *Altica* sp. |  |  |  |  |  |  | 1 |  |  |  |
| *Plagiosterna aenea* (Linnaeus, 1758) |  |  |  |  |  |  |  |  |  | 1 |
| **Curculionidae** |  |  |  |  |  |  |  |  |  |  |
| *Anisandrus dispar* (Fabricius, 1792) |  |  |  | 1 |  |  |  |  | 2 |  |
| *Bagous puncticollis* Boheman, 1845 |  |  |  |  |  |  |  | 1 |  |  |
| *Brachyderes incanus* (Linnaeus, 1758) |  |  |  | 1 |  |  | 1 |  |  |  |
| *Ips acuminatus* (Gyllenhal, 1827) | 1 |  |  |  |  |  |  |  |  |  |
| *Phyllobius arborator* (Herbst, 1797) |  |  |  |  |  |  |  |  | 1 |  |
| *Phyllobius maculicornis* Germar, 1823 |  |  | 1 |  |  |  |  |  |  |  |
| *Polydrusus cervinus* (Linnaeus, 1758) |  |  |  |  |  |  | 1 |  |  |  |
| *Strophosoma capitatum* (De Geer, 1775) | 1 |  |  | 1 |  |  |  |  |  |  |
| **LEPIDOPTERA** (unidentified specimens) | 512 | 1059 | 361 | 182 | 791 | 414 | 649 | 1245 | 829 | 1762 |
| **HYMENOPTERA** |  |  |  |  |  |  |  |  |  |  |
| **Vespidae** |  |  |  |  |  |  |  |  |  |  |
| *Ancistrocerus nigricornis* (Curtis, 1826) |  |  |  | 1 |  | 2 |  |  |  |  |
| *Ancistrocerus parietum* (Linnaeus, 1758) |  |  |  |  |  | 1 |  |  |  |  |
| *Discoelius zonalis* (Panzer, 1801) |  |  |  | 2 |  |  |  |  |  | 1 |
| *Dolichovespula media* (Retzius, 1783) | 8 | 53 |  | 2 |  | 4 | 12 | 39 | 23 | 24 |
| *Dolichovespula saxonnica* (Fabricius, 1793) |  |  |  | 1 |  | 1 |  |  |  | 3 |
| *Polistes nimpha* (Christ, 1791) |  |  | 2 |  |  |  |  |  |  | 4 |
| *Symmorphus murarius* (Linnaeus, 1758) |  |  | 1 |  | 3 | 5 |  |  |  |  |
| *Vespa crabro* Linnaeus, 1758 | 77 | 193 | 1 | 3 | 12 | 30 | 79 | 69 | 31 | 63 |
| *Vespula germanica* (Fabricius, 1793) | 3 | 3 | 8 | 2 | 1 | 2 | 1 |  | 1 | 96 |
| *Vespula rufa* (Linnaeus, 1758) |  |  |  | 1 |  |  |  |  |  |  |
| *Vespula vulgaris* (Linnaeus, 1758) | 31 | 53 | 18 | 13 | 10 | 49 | 10 | 19 | 14 | 238 |
| **Crabronidae** |  |  |  |  |  |  |  |  |  |  |
| *Ectemnius cephalotes* (Olivier, 1792) |  |  | 1 |  |  |  |  |  |  |  |
| *Diodontus medius* Dahlbom, 1844 |  |  |  |  |  |  | 1 |  | 2 |  |
| *Pemphredon inornata* Say, 1824 |  |  | 1 |  |  |  |  |  |  |  |
| *Pemphredon lugubris* (Fabricius, 1793) |  |  |  | 1 |  |  |  |  |  |  |
| *Psenulus pallipes* (Panzer, 1798) | 1 |  |  |  |  |  |  |  |  |  |
| **Chrysididae** |  |  |  |  |  |  |  |  |  |  |
| *Chrysis fulgida* Linnaeus, 1760 |  |  | 1 |  |  | 3 |  |  |  |  |
| *Chrysis ignita* (Linnaeus, 1758) | 1 |  |  |  |  |  |  |  |  |  |
| *Chrysis iris* Christ, 1791 |  |  |  |  |  | 1 | 1 |  |  |  |
| *Pseudomalus auratus* (Linnaeus, 1758) |  |  |  | 2 |  |  |  |  |  |  |
| *Pseudomalus pusillus* (Fabricius, 1804) |  |  |  |  |  |  | 1 |  |  |  |
| **DIPTERA** |  |  |  |  |  |  |  |  |  |  |
| **Anisopodidae** |  |  |  |  |  |  |  |  |  |  |
| *Sylvicola cinctus* (Fabricius, 1787) |  |  |  | 1 |  |  |  | 1 |  | 3 |
| *Sylvicola fuscatoides* (Michelsen, 1999) | 1 | 1 |  |  | 2 |  | 5 | 2 | 1 |  |
| *Sylvicola punctatus* (Fabricius, 1787) | 18 | 26 |  | 13 | 14 | 10 | 6 | 2 | 1 | 59 |
| *Sylvicola stackelbergi* Krivosheina & Menzel, 1998 | 1 |  |  |  |  |  |  | 1 |  |  |
| **Pallopteridae** |  |  |  |  |  |  |  |  |  |  |
| *Toxoneura saltuum* (Linnaeus, 1758) |  |  |  |  |  |  |  |  |  | 1 |
| *Toxoneura trimacula* (Meigen, 1826 ) |  |  |  |  |  |  |  | 1 |  |  |
| *Toxoneura usta* (Meigen, 1826) | 1 |  |  |  | 2 |  |  | 1 |  |  |
| **Platystomatidae** |  |  |  |  |  |  |  |  |  |  |
| *Platystoma lugubre* (Robineau-Desvoidy, 1830) | 58 | 53 | 66 | 12 | 114 | 110 |  | 1 |  | 121 |
| **Ulidiidae** |  |  |  |  |  |  |  |  |  |  |
| *Pseudotephritis millepunctata* (Hennig, 1939) | 7 | 26 |  |  | 2 | 1 | 4 | 4 | 1 | 1 |
| *Seioptera vibrans* (Linnaeus, 1758) |  |  |  |  |  |  |  |  |  | 1 |
| **Heleomyzidae** |  |  |  |  |  |  |  |  |  |  |
| Heleomyzidae sp. |  |  |  |  |  |  |  |  |  | 2 |
| *Suillia ustulata* (Meigen, 1830) | 1 |  |  |  |  |  |  | 1 | 1 |  |
| *Suilia* sp. |  |  |  |  |  |  |  | 11 | 3 | 14 |
| **Drosophilidae** |  |  |  |  |  |  |  |  |  |  |
| *Amiota albilabris* (Roth in Zetterstedt, 1860) | 2 | 1 |  |  |  |  |  |  |  |  |
| *Amiota alboguttata* (Wahlberg, 1839) |  | 7 |  |  |  |  |  |  |  | 2 |
| *Amiota rufescens* (Oldenberg, 1914) |  | 23 |  |  | 2 | 1 | 1 | 1 | 2 | 1 |
| *Amiota semivirgo* Maca, 1977 | 5 | 3 |  |  | 5 | 1 | 1 | 1 | 3 | 1 |
| *Chymomyza costata* (Zetterstedt, 1838) |  |  |  |  |  | 1 |  |  |  |  |
| *Drosophila bifasciata* Pomini, 1940 | 4 | 4 | 2 | 1 | 12 | 2 | 11 | 13 | 15 | 7 |
| *Drosophila histrio* Meigen, 1830 |  |  |  |  |  | 1 |  | 1 |  |  |
| *Drosophila obscura* Fallén, 1823 | 27 | 25 |  | 4 | 36 | 26 |  | 59 | 17 | 30 |
| *Drosophila phalerata* Meigen, 1830 |  | 1 |  |  |  |  |  |  |  |  |
| *Drosophila testacea* von Roser, 1840 |  |  |  |  | 2 | 1 |  | 2 |  |  |
| *Drosophila transversa* Fallén, 1823 |  |  |  |  | 1 |  |  |  |  | 1 |
| *Gitona distigma* Meigen, 1830 | 18 | 21 | 25 | 8 | 16 | 33 | 6 | 1 | 6 | 4 |
| *Leucophenga maculata* (Dufour, 1839) |  | 1 |  |  |  |  |  |  |  |  |
| *Leucophenga quinquemaculata* Strobl, 1893 | 7 | 7 |  | 1 | 3 |  | 5 | 6 | 5 | 7 |
| *Scaptodrosophila rufifrons* (Loew, 1873) |  | 8 | 1 | 1 | 2 | 1 | 1 | 13 | 3 | 16 |
| **Lonchaeidae** |  |  |  |  |  |  |  |  |  |  |
| *Lonchaea baechlii* MacGowan, 2016 | 1 | 2 |  |  |  |  |  |  | 1 |  |
| *Lonchaea carpathica* Kovalev, 1974 | 49 | 182 | 47 |  |  |  |  | 45 |  |  |
| *Lonchaea hackmani* Kovalev, 1981 |  | 1 |  |  |  |  |  |  |  |  |
| *Lonchaea limatula* Collin, 1953 | 12 | 74 | 14 |  | 1 | 2 | 1 | 5 |  |  |
| *Lonchaea sylvatica* Beling, 1873 |  | 1 |  |  |  |  |  |  |  |  |
| *Lonchaea xylophila* Kovalev, 1978 |  | 1 |  |  |  |  |  |  |  |  |
| *Protearomyia nigra* (Meigen, 1826) |  | 1 |  |  |  |  |  |  |  | 2 |
| *Protearomyia withersi* MacGowan, 2014 |  |  |  |  |  |  |  |  | 1 |  |
| **Syrphidae** |  |  |  |  |  |  |  |  |  |  |
| *Ferdinandea cuprea* (Scopoli, 1763) | 1 |  |  |  |  | 1 |  | 1 | 1 | 1 |
| *Volucella pellucens* (Linnaeus, 1758) |  |  |  |  |  | 1 |  |  | 1 | 1 |
| **Aulacigastridae** |  |  |  |  |  |  |  |  |  |  |
| *Aulacigaster leucopeza* (Meigen, 1830) |  |  |  |  |  |  |  |  | 1 | 1 |
| **Lauxaniidae** |  |  |  |  |  |  |  |  |  |  |
| *Meiosimyza decempunctata* (Fallén, 1820) |  |  |  |  |  |  |  |  | 1 |  |
| *Minettia lupulina* (Fabricius, 1787) | 1 | 2 |  |  | 2 |  |  | 4 |  | 3 |
| *Sapromyza schnabli* Papp, 1987 |  |  | 1 |  |  |  |  |  |  |  |
| **Dolichopodidae** |  |  |  |  |  |  |  |  |  |  |
| *Neurigona* sp. |  |  |  |  |  |  | 1 |  |  |  |
| **Anthomyiidae** |  |  |  |  |  |  |  |  |  |  |
| *Anthomyia* sp. |  | 2 | 1 | 1 | 1 | 3 |  |  |  | 3 |
| *Delia platura* (Meigen, 1826) | 8 | 1 | 13 | 16 | 17 | 10 | 3 |  | 1 | 1 |
| **Fanniidae** |  |  |  |  |  |  |  |  |  |  |
| *Fannia canicularis* (Linnaeus, 1760) | 3 | 3 | 1 |  | 2 | 2 | 1 | 1 | 2 | 1 |
| *Fannia sociella* (Zetterstedt, 1845) |  |  |  |  |  |  |  | 1 |  | 1 |
| *Fannia vespertilionis* Ringdahl, 1934 | 3 | 29 |  |  | 8 |  | 1 |  |  | 10 |
| **Muscidae** |  |  |  |  |  |  |  |  |  |  |
| *Achanthiptera rohrelliformis* (Robineau-Desvoidy, 1830) |  |  |  |  |  |  |  |  |  | 1 |
| *Helina evecta* (Harris, 1780) |  | 1 |  |  |  |  |  |  |  |  |
| Helina sp. | 53 | 158 | 16 | 5 | 47 | 73 | 38 | 176 | 201 | 507 |
| *Hydrotaea dentipes* (Fabricius, 1805) |  | 1 |  |  |  |  |  |  |  | 1 |
| *Mesembrina meridiana* (Linnaeus, 1758) |  |  |  |  |  |  |  |  |  | 4 |
| *Muscina levida* (Harris, 1780) | 1 | 4 |  |  | 2 |  |  |  |  |  |
| *Muscina prolapsa* (Harris, 1780) | 2 | 7 |  |  | 2 |  |  | 3 |  | 7 |
| *Phaonia fuscata* Fallén 1825 |  |  |  |  | 1 |  |  |  |  | 1 |
| *Phaonia pallida* (Fabricius, 1787) | 206 | 487 | 8 | 2 | 200 | 44 | 66 | 439 | 278 | 337 |
| *Phaonia rufiventris* (Scopoli, 1763) |  |  |  |  |  |  |  |  |  | 1 |
| *Thricops simplex* (Wiedemann, 1817) |  |  |  |  | 1 |  |  |  | 1 | 5 |
| **Calliphoridae** |  |  |  |  |  |  |  |  |  |  |
| *Pollenia* sp. | 41 | 43 | 20 | 8 | 22 | 18 | 8 | 9 | 7 | 97 |
| **MECOPTERA** |  |  |  |  |  |  |  |  |  |  |
| **Panorpidae** |  |  |  |  |  |  |  |  |  |  |
| *Panorpa* sp. |  |  |  |  |  |  |  | 2 | 2 | 37 |
| **TRICHOPTERA** |  |  |  |  |  |  |  |  |  |  |
| **Hydropsychidae** |  |  |  |  |  |  |  |  |  |  |
| *Hydropsyche* sp. |  |  |  |  |  |  |  | 1 |  | 3 |
